# Hydrogels containing redispersible spray-dried melatonin-loaded nanocapsules: a formulation for transdermal-controlled delivery

**DOI:** 10.1186/1556-276X-7-251

**Published:** 2012-05-15

**Authors:** Cristiane RD Hoffmeister, Taís L Durli, Scheila R Schaffazick, Renata P Raffin, Eduardo A Bender, Ruy CR Beck, Adriana R Pohlmann, Sílvia S Guterres

**Affiliations:** 1 Programa de Pós-Graduação em Ciências Farmacêuticas, Universidade Federal do Rio Grande do Sul, Av. 2752, Porto Alegre, CEP, 90610-000, Brazil; 2 Departamento de Farmácia Industrial, Centro de Ciências da Saúde, Universidade Federal de Santa Maria, Prédio 26 - Campus UFSM, Santa Maria, CEP 97105-900, Brazil; 3 Programa de Pós-Graduação em Nanociência, Centro Universitário Franciscano, Rua dos Andradas, 1614, Santa Maria, 97010-032, Brazil; 4 Departamento de Química Orgânica, Universidade Federal do Rio Grande do Sul, CP 15003, Porto Alegre, CEP 91510-970, Brazil

**Keywords:** Hydrogels, Maltodextrin, Melatonin, Nanocapsules, Spray-drying, Lactose, Skin permeation, Transdermal delivery

## Abstract

The aim of the present study was to develop a transdermal system for controlled delivery of melatonin combining three strategies: nanoencapsulation of melatonin, drying of melatonin-loaded nanocapsules, and incorporation of nanocapsules in a hydrophilic gel. Nanocapsules were prepared by interfacial deposition of the polymer and were spray-dried using water-soluble excipients. *In vitro* drug release profiles were evaluated by the dialysis bag method, and skin permeation studies were carried out using Franz cells with porcine skin as the membrane. The use of 10% (*w*/*v*) water-soluble excipients (lactose or maltodextrin) as spray-drying adjuvants furnished redispersible powders (redispersibility index approximately 1.0) suitable for incorporation into hydrogels. All formulations showed a better controlled *in vitro* release of melatonin compared with the melatonin solution. The best controlled release results were achieved with hydrogels prepared with dried nanocapsules (hydrogels > redispersed dried nanocapsules > nanocapsule suspension > melatonin solution). The skin permeation studies demonstrated a significant modulation of the transdermal melatonin permeation for hydrogels prepared with redispersible nanocapsules. In this way, the additive effect of the different approaches used in this study (nanoencapsulation, spray-drying, and preparation of semisolid dosage forms) allows not only the control of melatonin release, but also transdermal permeation.

## Background

Melatonin (*N*-acetyl-5-methoxytryptamine) is a hormone secreted by the pineal gland of mammals at nighttime, and it is involved in the regulation of the sleep-wake cycle as well as in several biological functions, including the regulation of mood, the control of seasonal reproduction, and the circadian rhythm, in animals and humans. The administration of dosage forms containing exogenous melatonin has been used to treat circadian rhythm disorders such as jet lag and insomnia [1]. Besides being a potent antioxidant, melatonin is a free scavenger [1,2]; it protects against lipid peroxidation in several models [3] and against oxidative stress in some neurodegenerative diseases such as Alzheimer's disease [1,4]. Furthermore, it protects against ischemia/reperfusion injury [5] and has antitumor activity [6].

Melatonin has a very short half-life and low and variable oral bioavailability presumably due to variable degrees of absorption and/or an extensive metabolism by the liver [7,8]. Furthermore, melatonin is a poorly water-soluble substance and has slow dissolution characteristics [9]. Hence, melatonin is not a good candidate for conventional oral immediate-release dosage forms [8]. Thus, melatonin-sustained release formulations have been developed for oral [8-14], intranasal [15], transdermal [16-18], and transmucosal [18,19] administrations. Modified release tablets containing melatonin, including β-cyclodextrin [9], microparticles [8,13,15], hydroxypropylmethylcellulose matrix tablets [7], and lecithin/chitosan nanoparticles [19], have been also successfully prepared. Transdermal administration of melatonin could be a very attractive alternative resulting in sustained drug plasma levels that can be tailored to the normal physiological range and avoid the first pass effect [16]. Additionally, transdermal delivery could overcome the low oral bioavailability of melatonin [20]. In this context, the skin protecting and non-irritant nature of melatonin supports its candidature for transdermal delivery [17].

Over the past few decades, there has been considerable interest in studying polymeric nanostructures as effective drug delivery devices. Nanocapsules are vesicular systems composed of an oil-filled cavity surrounded by a polymeric wall presenting narrow particle size distribution, and they are stabilized by surfactants at the particle/water interface [21-23]. In nanocapsules (<1 μm), the drug can be dissolved, entrapped, encapsulated, and/or adsorbed or attached to the polymeric particles [21,22]. Despite the huge variety of promising applications of polymeric nanocapsules, in the area of transdermal delivery, these carriers have not yet been explored. This *via* is advantageous because it is minimally invasive, therefore avoids pain and risk of infections.

Previous studies have demonstrated that melatonin-loaded Eudragit® S100 nanocapsule suspensions were able to improve the antioxidant effect of melatonin in both *in vitro* and *in vivo* experiments [24,25]. In some cases, these aqueous suspensions present limited physicochemical stability under storage due to the possibility of particle aggregation [26], the degradation of components such as the polymer [27-29] or drug leakage [29-31]. Freeze-drying [23,29] and spray-drying techniques [32,33] have been reported as alternatives to improve the stability of nanoparticle suspensions by means of their conversion into powders. Spray-dried powders containing melatonin-loaded Eudragit® S100 nanocapsules were prepared using silicon dioxide as drying adjuvants, showing a controlled drug release profile in comparison with the pure drug [34]. In addition, redispersible drug-free spray-dried poly(ϵ-caprolactone)-nanocapsules were developed using lactose as the excipient [35].

Although the results of previous studies have demonstrated the feasibility of preparing redispersible dried polymeric nanocapsules, there is a lack of information in the literature on the use of these powders for the development of nanomedicines or nanocosmetics as final dosage forms. In this context, the aim of this study was to control the *in vitro* transdermal delivery of melatonin by means of a strategy based on hydrogel formulations containing spray-dried melatonin-loaded nanocapsules. We hypothesized that the nanocarriers dried with the aid of a water-soluble excipient and incorporated into the semisolid formulation could modulate the transdermal delivery of melatonin.

## Methods

### Materials

Melatonin was obtained from Acros Organics (Geel, Belgium). Poly(methacrylic acid-co-methyl methacrylate) (Eudragit S100®) was supplied by Almapal (São Paulo, Brazil). The caprylic/capric triglyceride mixture was obtained from Brasquim (Porto Alegre, Brazil). Sorbitan monooleate (Span 80®), polysorbate 80 and triethanolamine were acquired from Delaware (Porto Alegre, Brazil). Carbopol 940® was obtained from BF Goodrich (Charlotte, NC, USA). Lactose and maltodextrin were purchase from Henrifarma (São Paulo, Brazil) and Roquette (Lestrem, France), respectively. All other chemicals and solvents were of pharmaceutical grade and used as received.

### Preparation and characterization of nanocapsule suspensions

Melatonin-loaded polymeric nanocapsules (NC) were prepared (*n* = 3) by interfacial deposition of the preformed polymer according to the method described by Fessi and co-workers [36]. The organic phase consisted of melatonin (0.0125 g), caprylic/capric triglyceride mixture (0.8 mL), Eudragit S100® (0.25 g), and Span 80® (0.1915 g) in acetone. This lipophilic solution was poured into a hydrophilic phase containing polysorbate 80 (0.1915 g). Acetone was removed, and the suspensions were concentrated by evaporation under reduced pressure to obtain a final volume of 25 mL (0.5 mg mL^−1^ of melatonin) [24].

The mean size and polydispersity of the nanocapsules were determined at 25 ± 2°C by photon correlation spectroscopy (Zetasizer Nano ZEN3600, Malvern, UK). Suspensions were diluted 500-fold in MilliQ® water (Millipore Co., Billerica, MA, USA). Zeta potential was measured by an eletrophoretic technique, using the same equipment. For these measurements, the suspensions were diluted (1:500) with 10 mM NaCl aqueous solution. The pH measurements were carried out directly in the samples using a Micronal B474 potentiometer (Micronal, São Paulo, Brazil).

The melatonin content of the nanocapsules was determined after their dissolution in acetonitrile and assayed by high-performance liquid chromatography (HPLC) [24]. The system consisted of an SPD-10A Shimadzu detector, LC-10 AD Shimadzu pump, SIL-10A Shimadzu injector (Shimadzu Corporation, Nakagyo-ku, Kyoto, Japan), and Lichrospher® RP-18 column provided by Merck (Darmstadt, Germany). The mobile phase consisted of acetonitrile/water (55:45, *v*/*v*) at a flow rate of 0.7 mL min^−1^. Melatonin was detected at 229 nm. The encapsulation efficiency (percentage) was calculated by difference between the total content and free melatonin concentration in the nanocapsule suspension. The free melatonin concentration was determined using the ultrafiltration-centrifugation technique (Microcon 10,000 KDa, Millipore) [24].

### Preparation and characterization of spray-dried polymeric nanocapsules

Spray-dried melatonin-loaded nanocapsules were prepared (*n* = 3) using an MSD® 1.0 spray dryer (Labmaq, São Paulo, Brazil). Lactose and maltodextrin at 10% (*w*/*v*) were evaluated individually as drying adjuvants. The drying adjuvant was added to the nanocapsule suspension under magnetic stirring. The stirring was maintaining for 10 min before the formulation was fed into the spray dryer. A two-fluid nozzle with a cap orifice diameter of 0.7 mm and a co-current flow was used. The inlet temperature in the drying chamber was maintained at 150 ± 10°C, and the feeding rate was set at 0.3 L/h [35]. Powders prepared with lactose and maltodextrin were named as D-NC-L and D-NC-M, respectively.

The process yield (percentage) was calculated as the ratio between the total weight of powder recovered in the sample collector and the total dry mass of the components used. The residual moisture content (percentage) of each spray-dried product was measured by Karl Fischer titration in dry methanol (Titro Matric 1 S, Crison Instruments, Barcelona, Spain). Measurements were performed in triplicate. In order to determine the melatonin content in the spray-dried powders, samples were dispersed in methanol and kept under magnetic stirring for 10 min. After centrifugation, melatonin was assayed by HPLC according to the method described above.

The size of the spray-dried powder particles was measured by laser diffractometry (Mastersizer 2000®, Malvern, UK). Measure of the width of the distribution of particle size (SPAN) values were used, and they were calculated by (*d*_0.9_ − *d*_0.1_)/*d*_0.5_ where *d*_0.9_, *d*_0.5_, and *d*_0.1_ are the particle diameters determined respectively at the 90th, 50th, and 10th percentile of undersized particles. The deagglomeration profile of the dried particles was evaluated as a function of time after their dispersion in water (Hydro SM small volume sample dispersion unit, Malvern, UK). Aliquots were analyzed every 5 min for 60 min. The aliquots acquired from 0 and 60 min were also centrifuged, filtered (0.45 μm, Millipore), and analyzed by photon correlation spectroscopy (Zetasizer Nano Series ZEN3600, Malvern, UK). Furthermore, nanoparticle tracking analysis (NanoSight LM10 and NTA 2.0 Analytical Software, NanoSight, Wiltshire, UK) was also used as a tool to estimate the particle size distribution and to gain visual information. This experiment was carried out using 0.5 mL of the diluted samples (1:5,000 in milliQ water *v*/*v*) introduced into the chamber by means of a syringe. The chamber was placed on the optical microscope, and the particles were illuminated by a laser diode (635-nm wavelength). The video images of the Brownian motion of the individual particles were obtained real time via CCD camera and analyzed using the NTA 2.0 Analytical Software (NanoSight, UK). In the nanoparticle tracking analysis (NTA) method, the particle sizing system is based on the scattering of light, where all visible particles in the sample and each separate light scattering center are seen as an individual particle during filming. Each video clip was captured over 120 s. The automatic detection threshold was enabled, and the maximum particle jump was set at 10 in. the NTA software. The size values obtained by NTA were used to evaluate the redispersion efficiency (RE) of the spray-dried systems. The RE was calculated using Equation 1, where values close to one indicate a narrow redispersion of the powders

(1) $$\text{RE}=\frac{d_{\text{RP}}}{d_{\text{S}}}\text{,}$$

where *d*_RP_ is the mean particle size of the redispersed spray-dried powder and *d*_S_ is the mean particle size of the original nanocapsule suspension.

Morphological analysis of spray-dried powders was carried out by scanning electron microscopy (JEOL scanning microscope JSM-5800, Tokyo, Japan). Samples were analyzed after they had been gold sputtered (JEOL Jee 4B SVG IN, Tokyo, Japan), and the analysis was performed at the Electron Microscopy Center of the Federal University of Rio Grande de Sul State (Centro de Microscopia Eletrônica - UFRGS).

### Preparation and characterization of hydrophilic gels

Hydrogels were prepared using 0.5% of Carbopol 940®, 0.2% of diazolinidyl urea, and triethanolamine. Four different formulations were prepared: (a) a hydrogel prepared by adding Carbopol 940® directly to the nanocapsule suspension (G-NC), (b) a hydrogel prepared by adding the spray-dried nanocapsules (prepared with lactose) to a preformed hydrogel (G-NC-L), (c) a hydrogel prepared by adding the spray-dried nanocapsules (prepared with maltodextrin) to a preformed hydrogel (G-NC-M), and (d) a hydrogel prepared by adding directly the melatonin dispersed in water containing polysorbate 80 at 0.77% to preformed hydrogels (G-M). All formulations were prepared at a final concentration of 0.5 mg g^−1^ of melatonin. Hydrogels containing redispersed spray-dried nanocapsules (G-NC-L and G-NC-M) were prepared by adding 1.57 g of powder in 10 g of each hydrogel previously prepared, reaching a final concentration of 0.5 mg g^−1^ of melatonin. The pH values of the gels were determined using a potentiometer (Micronal B474, São Paulo, Brazil) through the direct immersion of the electrode in semisolids (*n* = 3). The physical stability of the hydrogels was evaluated by multiple light scattering (Turbiscan Lab, Formulaction, L'Union, France). The samples were poured into glass cells without any dilution and analyzed using scan mode every 6 min for 20 h at room temperature.

### *In vitro* drug release profiles

*In vitro* drug release profiles (*n* = 3) from all formulations (nanocapsule suspensions, dried nanocapsules, and hydrogels) were studied under sink conditions using a cellulose dialysis bag (MWCO = 12,000 to 14,000 Da, Sigma-Aldrich Corporation, St. Louis, MO, USA). Nanocapsule suspensions (10 mL) and the hydrogels (10 g) were transferred directly to the dialysis bags, which were placed in a beaker containing 150 mL of 5% polysorbate 80 aqueous solution (at 37°C) with slow magnetic stirring. For the spray-dried nanocapsules, the powders were previously redispersed in 10 mL of water. The initial concentration of melatonin in the dialysis bag was 0.5 mg mL^−1^ for all samples. Aliquots of 2 mL were withdrawn periodically and replaced with the same volume of fresh medium. The concentration of melatonin released at each time was determined by HPLC using a validated methodology previously described [24]. Drug release profiles were analyzed by model-dependent methods (mono-exponential and bi-exponential models) using the software MicroMath Scientist® (St. Louis, MO, USA). The best model to describe the release profile was selected based on the highest model selection criterion (MSC) and the highest correlation coefficient (*r*), as well as the best curve fitting.

### *In vitro* skin permeation studies

The *in vitro* permeation experiments were performed using Franz type diffusion cells and pig abdomen skin at 37°C. Pig skin was obtained from a local slaughterhouse. The skin was cleaned to remove the hair and adipose tissue and kept at −20°C until use. The thickness of the skin piece (1.5 to 2 mm) was measured using a micrometer (Dial Thickness Gage, 2046 S, Mitutoyo Corporation, Kanagawa, Japan). The dermal side of the porcine flank skin was exposed to the receptor fluid (5.0% (*v*/*v*) polysorbate 80 aqueous solution), and the stratum corneum was exposed to the air (non-occlusive conditions). The effective permeation area was 16 cm^2^, and the volume of the receiver chamber was around 50 mL.

The hydrogels were applied and weighed in the donor compartment (50 mg/cm^2^). The flux of melatonin from the nanocapsules through the skin was calculated by determining the drug concentration in the receptor medium at predetermined times (2, 4, 6, 8, 12, and 24 h) by liquid chromatography (as previously described). Results represent the mean of six independent replicates (*n* = 6). The cumulative amounts of melatonin per diffusion area were calculated for each time point and plotted versus the sampling time points. Flux corresponds to the slope calculated by the linear regression of these data points.

### Statistical analysis

All analyses were carried out in triplicate. Results are expressed as the mean ± standard deviation. Parameters of the mathematical modeling (*k*, *A*, *α*, *B*, and *β*) and the data of *in vitro* melatonin permeation were statistically evaluated using one-way analysis of variance at a significance level of 5% (StatGraphics Plus 5.1, STATPOINT TECHNOLOGIES, INC., Warrenton, VA, USA).

## Results and discussion

### Polymeric nanocapsule suspensions

The melatonin-loaded nanocapsule suspension revealed a mean particle size of 186 ± 24 nm with a low polydispersity index (0.16 ± 0.03) and a pH of 3.87 ± 0.14. The zeta potential was negative (−11.2 ± 5.1 mV) because of the negative surface charge density at the particle/water interface as a consequence of the presence of oxygen atoms in the polymer backbone [37,38]. Drug content in the final nanocapsule suspension was 0.477 ± 0.003 mg ml^−1^ with an encapsulation efficiency of 63 ± 2%. All these results are in accordance with the method of preparation, drug and materials used, as previously reported by our group [24].

### Spray-dried polymeric nanocapsules

Nanocapsule suspensions were spray-dried using lactose or maltodextrin. The process yields were of 52 ± 8% and 50 ± 6% for spray-dried formulations using lactose (D-NC-L) or maltodextrin (D-NC-M), respectively. The operational conditions of the spray-drying process were appropriate for the drying of the nanocapsules, as demonstrated by the low moisture content of the dried formulations (1.1 ± 0.1% and 1.7 ± 0.3% for D-NC-L and D-NC-M, respectively).

Melatonin content in the spray-dried nanocapsules was 3.17 and 3.36 mg g^−1^, for D-NC-L and D-NC-M, respectively, corresponding to a complete recovery of the drug after the drying process (99% and 105%, respectively). However, some previous reports have described that a segregation of the drug/materials during the spray-drying process can occur, depending on the raw material [33] or the parameters of the process [39].

**Figure 1:**
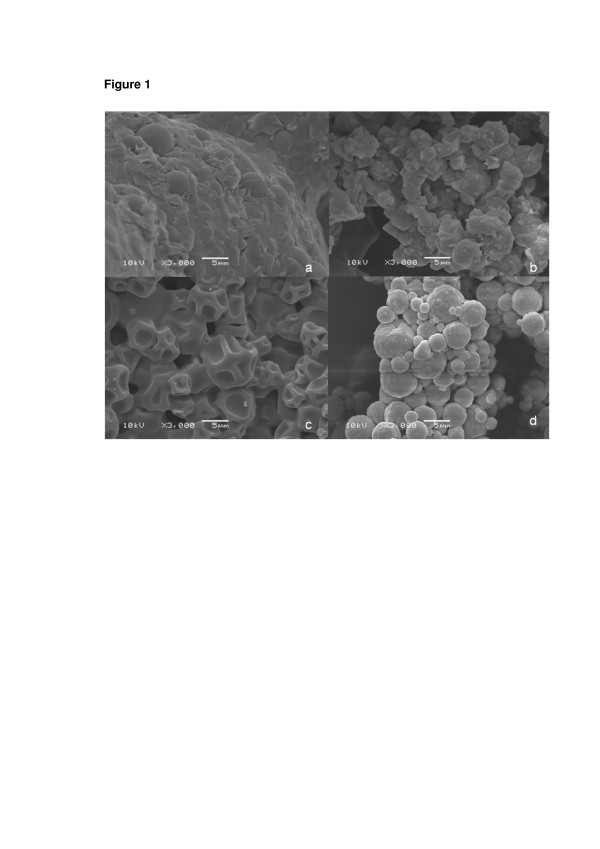
**Images obtained by SEM of powders.** (**a**) Pure lactose, (**b**) dried nanocapsules using lactose (D-NC-L), (**c**) pure maltodextrin, and (**d**) dried nanocapsules using maltodextrin (D-NC-M) at × 3,000.

**Figure 2:**
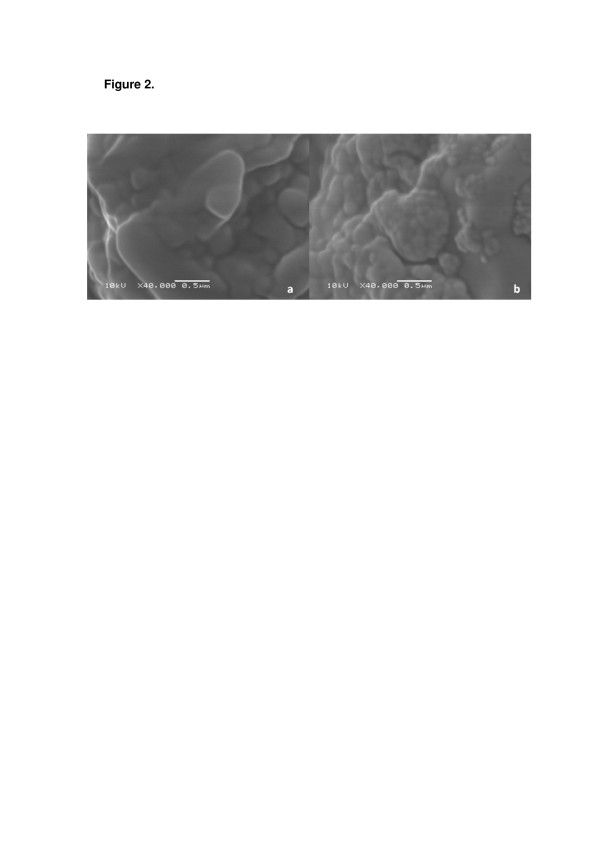
**Images obtained by SEM of powders.** (**a**) D-NC-L and (**b**) D-NC-M at × 40,000.

Scanning electron microscopy (SEM) analysis showed that the morphologies of the adjuvants and the spray-dried nanocapsules vary considerably (Figure 1). Dried lactose showed large agglomerates (Figure 1a), while the spray-dried nanocapsules (D-NC-L) demonstrated rugged microagglomerates (Figure 1b). On the other hand, dried maltodextrin displayed collapsed microballoons (Figure 1c). Spray-dried nanocapsules (D-NC-M) showed rugged spherical microagglomerates (Figure 1d) similar to those observed for dried powders prepared using lactose. At higher magnification (×40,000), nanocapsules can be observed in both microagglomerates (Figure 2).

**Figure 3:**
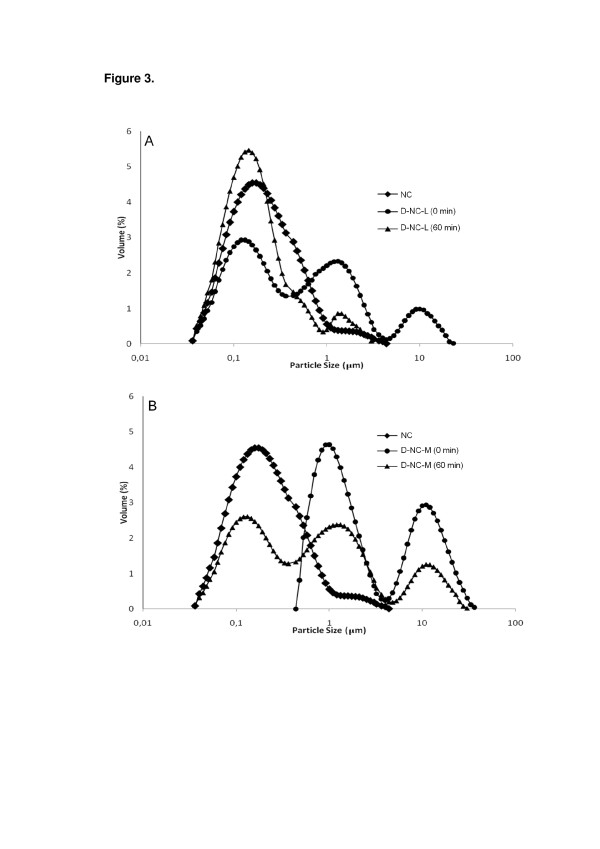
**Particle size distribution.** (**a**) NC and D-NC-L at 0 and 60 min, and (**b**) NC and D-NC-M at 0 and 60 min.

Water-soluble adjuvants were selected to dry melatonin-loaded nanocapsules in order to obtain high redispersion efficiency. Samples of redispersed D-NC-L and D-NC-M were analyzed by laser diffractometry. Particle size distributions were determined based on equivalent sphere approximation after dispersing the powders in water (0 and 60 min). Particle size distribution profiles of redispersed D-NC-L and D-NC-M were compared to that of the original nanocapsule suspension (Figure 3). Immediately after dispersion, the D-NC-L had three main particle populations. After 60 min, the profile showed the disappearance of the population corresponding to the higher particle size, a decrease in the intermediary particle size population, and an increase in the nanometric population. This nanometric population presented a profile similar to that of the original nanocapsule suspension, verifying that the D-NC-L system can be redispersed, reaching nanosize distribution. In the case of the D-NC-M powder, the profile showed two micrometric populations immediately after redispersion in water. After 60 min, the areas of these populations decreased, and a nanometric population was observed. Although a micrometric population can still be observed, the nanoscopic population presented a profile similar to that of the original nanocapsule suspension. This result indicated that D-NC-M system can be partially redispersible, reaching nanosize distribution.

**Figure 4:**
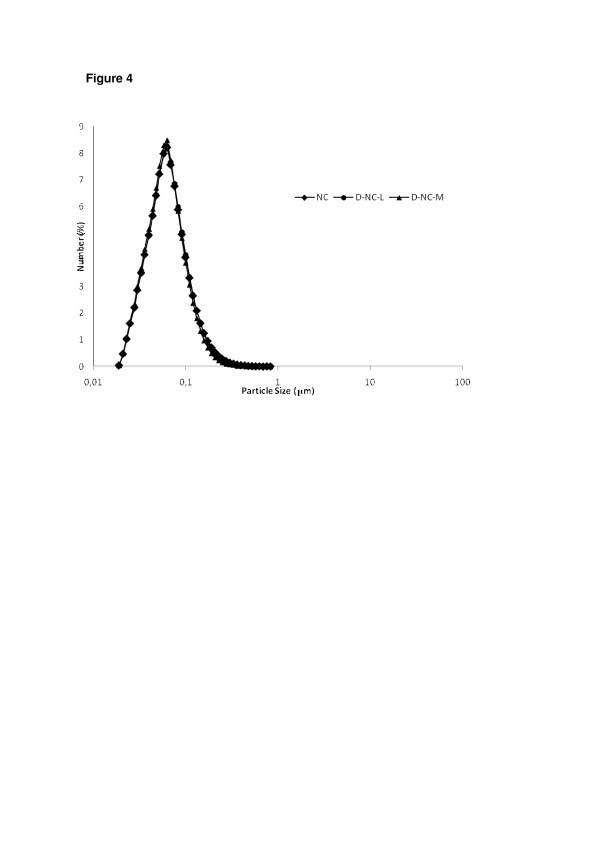
**Particle size distribution (as number of particles) after 60 min of aqueous redispersion.** (NC, D-NC-L, and D-NC-M).

Considering that there is a cubic relationship between the size of a particle and its volume, we also analyzed the size distribution in terms of numbers of particles (Figure 4). After 60 min, D-NC-L and D-NC-M presented similar particle size profiles as that of the original suspension (NC). Thus, the contribution of the micrometric populations observed for both redispersed powders after 60 min can be considered negligible.

To fully characterize the nanoscopic population, formulations were also analyzed by photon correlation spectroscopy. D-NC-L and D-NC-M showed *z*-averaged diameters of 192 ± 50 nm and 181 ± 27 nm with polydispersity indexes of 0.33 ± 0.03 and 0.25 ± 0.10, respectively. In order to determine the redispersibility efficiency, the values for the ratio between the mean diameter of D-NC-L or D-NC-M and the mean size of the original nanocapsules were calculated. The results obtained were close to unity (1.03 and 0.97 for D-NC-L and D-NC-M, respectively), demonstrating that the dehydration-rehydration process did not lead to significant changes in the nanometric size of the systems, regardless of the adjuvant added.

**Figure 5:**
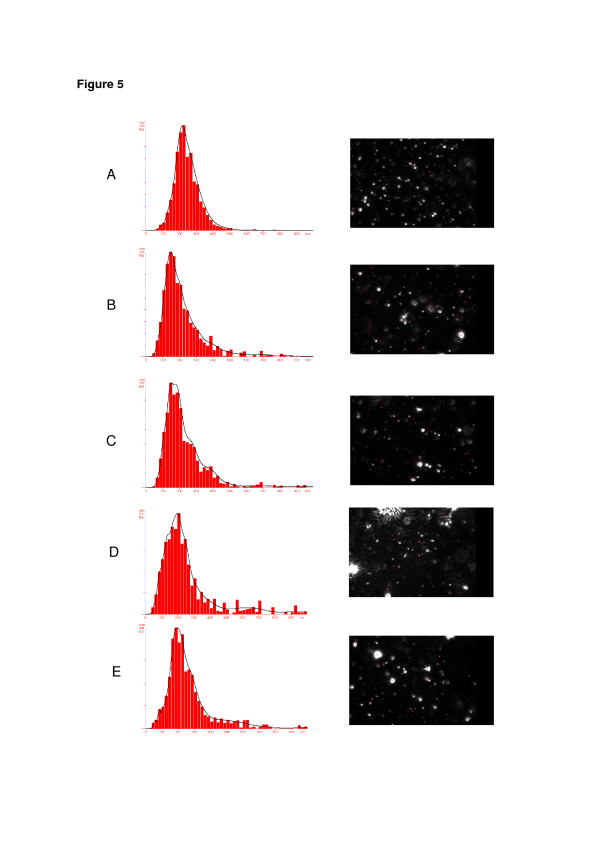
**Particle size distribution (nanometers) (left) and sample video frame (right) obtained from NTA.** (**a**) Original NC; (**b**) redispersed D-NC-L at time 0; (**c**) redispersed D-NC-L after 60 min; (**d**) redispersed D-NC-M at time 0; and (**e**) redispersed D-NC-M after 60 min.

Besides photon correlation spectroscopy (PCS), a new technique based on the particle diffusion was used to confirm the redispersion efficiency by determining the recovery of the nanoscopic sizes after the aqueous redispersion of the powders. NTA is based on a microscopic view of the particles illuminated by a laser diode. Different intensities of the scattered light are indicative of different particle sizes as larger particles scatter higher intensity light, and hence, larger luminous dots are observed [40,41]. On the other hand, smaller particles move faster and over longer distances with respect to larger particles [40]. The particle size distributions of the original nanocapsule suspension, D-NC-L, and D-NC-M were determined by NTA after 0 and 60 min. Figure 5 presents the particle size distributions and a video frame of each sample. The melatonin-loaded nanocapsule suspension had an average particle size of 249 nm with a SPAN value of 0.75 (Figure 5a). Immediately after redispersion of the powders in water, the D-NC-L had a mean particle size of 228 nm with a SPAN value of 1.43 (Figure 5b). The presence of lactose as an adjuvant probably led to the appearance of a few particles with sizes between 600 and 900 nm. In the case of D-NC-M, the mean particle size was 280 nm with a SPAN value of 2.06 (Figure 5d), indicating a broader distribution compared with D-NC-L. Thus, lactose as an adjuvant furnished a powder that is presenting better homogeneity immediately after redispersion in water. After 60 min of dispersing the powders, D-NC-L and D-NC-M showed average sizes of 261 and 277 nm with SPAN values of 1.43 and 1.44, respectively (Figure 5c,e). D-NC-L and D-NC-M presented similar particle size distributions after 0 and 60 min compared to the nanocapsule suspension. The calculated redispersibility efficiencies were 0.92 and 1.05 (D-NC-L) and 1.12 and 1.11 (D-NC-M), respectively. The results corroborate the findings obtained by PCS analysis.

### Hydrogels containing spray-dried melatonin-loaded nanocapsules

Melatonin-loaded nanocapsule suspensions and spray-dried nanocapsules (D-NC-L and D-NC-M) were incorporated into semisolid hydrogels (G-NC, G-NC-L, and G-NC-M, respectively). Hydrogels were white and bright with pH values being close to neutral for G-NC-L (7.11 ± 0.02) and for G-NC-M (7.10 ± 0.01); while for G-NC, a slightly acid pH was observed (5.8 ± 0.04).

**Figure 6:**
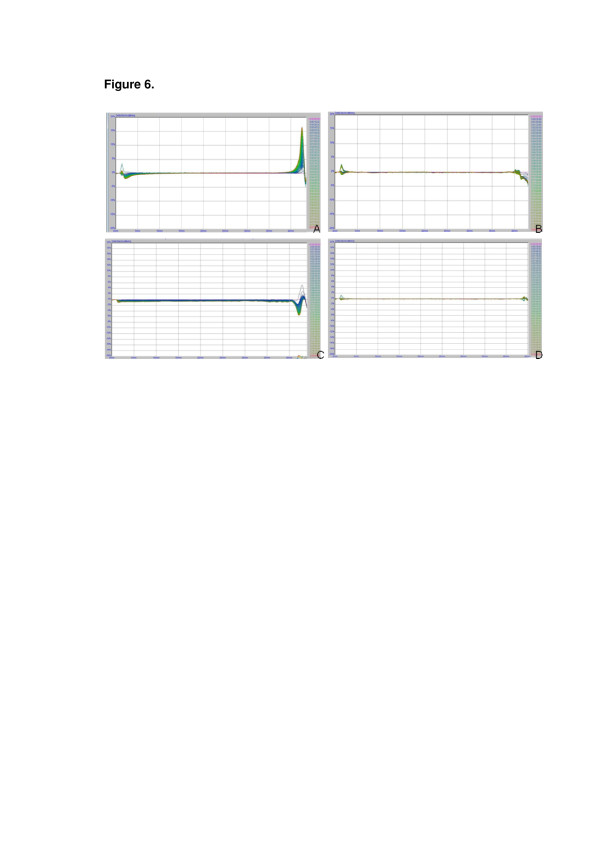
**Backscattering profiles of formulations.** (**a**) NC, (**b**) G-NC, (**c**) G-NC-M, and (**d**) G-NC-L.

The physical stability of the hydrogels was determined by multiple light scattering (Figure 6). In each graph, the left side corresponds to the bottom of the glass cell, and the right corresponds to the top. As a control, the nanocapsule suspension (NC) was also studied (Figure 6a). This suspension showed a slight increase in the backscattering at the top, associated with the creaming phenomenon, which is a reversible type of physical instability. After incorporating the nanocapsule suspension into a hydrogel (G-NC), the creaming disappeared because of the higher viscosity of the hydrogel, which reduces the particle mobility (Figure 6b). Regarding G-NC-M, a decrease in the relative backscattering at the center of the cell was observed (Figure 6d), indicating a decrease in particle size as a function of time. On the other hand, no changes were observed in the analysis of G-NC-L (Figure 6c), indicating a better physical stability compared with G-NC-M. All variations in the relative backscattering were lower than 5%, demonstrating a good physical stability of formulations.

### *In vitro* melatonin release studies

**Figure 7:**
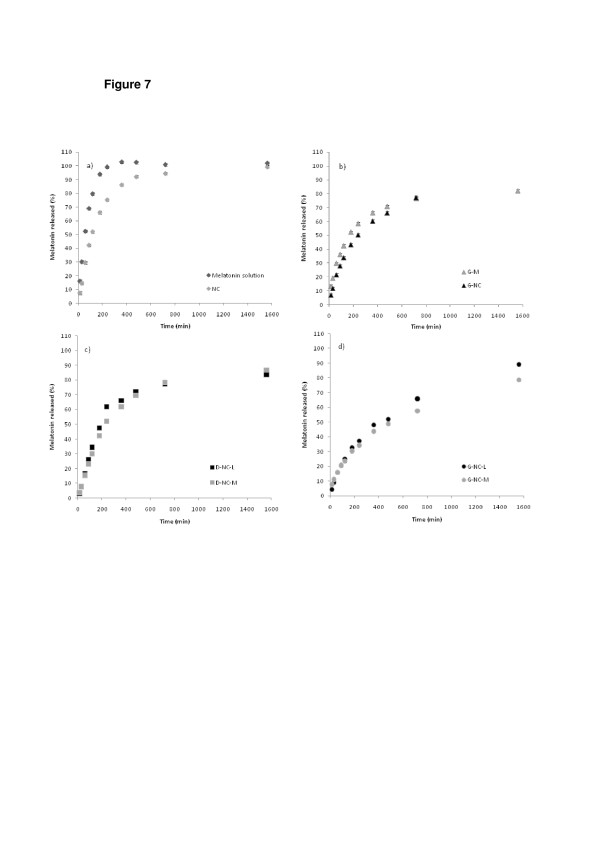
**Profiles of melatonin release.** These profiles are from (**a**) nanocapsule suspension in comparison to the melatonin solution, (**b**) hydrogels (G-M or G-NC), (**c**) spray-dried powders (D-NC-L and D-NC-M), and (**d**) hydrogels prepared with spray-dried powders (G-NC-L and G-NC-M).

The melatonin release from the nanocapsules was 90% after 480 min, while the diffusion of the drug from the solution reached 100% in 240 min (Figure 7a). The incorporation of either melatonin in solution or the melatonin-loaded nanocapsule suspension in hydrogels (G-M or G-NC, respectively) caused a decrease in the melatonin released within these time intervals (61% and 63%, respectively) as well as in the standard deviations (Figure 7b). These differences can be explained by the higher viscosity of the gels compared with the liquid formulations (NC and melatonin solution). Regarding the spray-dried powders, after 480 min D-NC-L and D-NC-M released 60% and 47%, respectively (Figure 7c), and G-NC-L and G-NC-M released 47% and 42%, respectively (Figure 7d). The release behavior of the hydrogels compared to the powders and the liquid formulations is a consequence of three contributions: (a) nanoencapsulation of melatonin, (b) microagglomeration of nanocapsules in the presence of the drying adjuvant, and (c) incorporation in a semisolid vehicle (Carbopol® hydrogel).

The release profiles of NC, D-NC-L, D-NC-M, and their respective hydrogels, G-NC, G-NC-L, and G-NC-M, were modeled using the mono- or bi-exponential model. The model that best fitted the experimental data was selected based on the highest correlation coefficient (*r*), highest MSC, and the best curve fitting. Thus, the bi-exponential was the best model to describe the release profile for all formulations, with correlation coefficients higher than 0.99 (Table 1). The kinetic parameters *A* were higher than 0.70 for NC, D-NC-L, and D-NC-M, suggesting that melatonin release was driven by a burst effect. Release rates of the burst and sustained phases (*k*_1_ and *k*_2_) calculated for the dried formulations were lower than that obtained for the nanocapsule suspension (NC). The results indicated that melatonin release from the powders was slower than from the liquid dosage forms. Additionally, the hydrogels (G-NC, G-NC-L, G-NC-M) showed kinetic parameters *B* that are higher than 0.70 (Table 1). Therefore, melatonin was mostly released in the second phase, indicating a sustained drug release. Thus, the addition of these formulations in semisolid vehicles reduced the burst release of melatonin, leading to prolonged release over time.

**Table 1:** Calculated release parameters of different formulations containing melatonin-loaded nanocapsules, according to the bi-exponential model

**Formulation**	**Parameter**			
	***A***	***B***	***k***_**1**_**(min**^**−1**^**)**	***k***_**2**_**(min**^**−1**^**)**
NC	0.88 ± 0.10	0.15 ± 0.11	0.0073 ± 0.0005	0.0017 ± 0.0011
D-NC-L	0.80 ± 0.02	0.28 ± 0.00	0.0058 ± 0.0002	0.0003 ± 0.0000
D-NC-M	0.78 ± 0.01	0.24 ± 0.02	0.0043 ± 0.0004	0.0004 ± 0.0001
G-NC	0.27 ± 0.04	0.72 ±0.04	0.0120 ± 0.0011	0.0016 ± 0.0001
G-NC-L	0.16 ±0.01	0.84 ± 0.00	0.1647 ± 0.0012	0.0012 ± 0.0000
G-NC-M	0.21 ± 0.10	0.74 ± 0.01	0.0072 ± 0.0002	0.0008 ± 0.0000

### *In vitro* permeation studies

**Figure 8:**
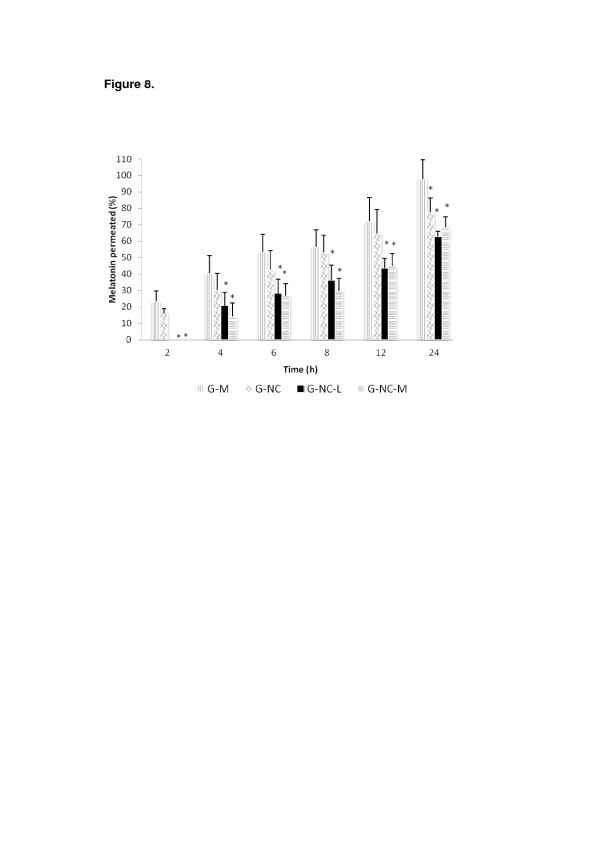
***In vitro*permeation profiles of melatonin from hydrogels.** G-NC, original nanocapsules; G-NC-L and G-NC-M, dried nanocapsules; and G-M, non-encapsulated melatonin (*significance, *p* ≤ 0.05).

The skin permeation of melatonin from hydrogels (G-M, G-NC, G-NC-L, and G-NC-M) was studied using Franz cells with porcine skin as the membrane. Melatonin permeated into the receptor compartment was measured at different time intervals (2 to 24 h; Figure 8). After 2 and 24 h, 25% and 100% of the melatonin from G-M permeated through the skin, respectively, and for G-NC, these values were 20% and 75%, respectively. No statistical differences (*p* > 0.05) in the permeated melatonin were observed in comparing G-NC and G-M for up to 12 h of the experiment. On the other hand, after 24 h, the melatonin permeation was lower for G-NC compared with that of G-M (*p* ≤ 0.05), showing the influence of nanoencapsulation on the permeation. In addition, a lower drug flux was observed for G-NC compared with G-M (0.014 ± 0.002 μg cm^−2^ h^−1^ and 0.018 ± 0.002 μg cm^−2^ h^−1^, respectively; *p* ≤ 0.05). Formulations containing spray-dried powders showed lower amounts of permeated melatonin for all time periods (20% and 15% of melatonin for G-NC-L and G-NC-M, respectively after 4 h) compared with those of G-M or G-NC (*p* ≤ 0.05), regardless of the type of the drying adjuvant. This result may be explained by the higher control of mela-tonin release from such formulations compared to the melatonin-loaded nanocapsules due to the presence of lactose or maltodextrin. Furthermore, lower drug fluxes were observed forG-NC-L and G-NC-M (0.011 ± 0.002 and 0.013 ± 0.002 ug.cm^-2^.h^-1^) compared with the hydrogel prepared with non-encapsulated melatonin (G-M, 0.018 ± 0.001 μg cm^−2^ h^−1^). However, on comparing the drug fluxes from G-NC-L and G-NC-M with those from G-NC, no statistical differences were observed. The presence of lactose or maltodextrin caused a delayed permeation of melatonin across the skin as observed in Figure 8 (after 2 and 4 h of experiment). The influence on these results of the rheological properties of the hydrogels in the presence of these adjuvants should be evaluated in further studies.

In general, the results of the permeation study corroborate those obtained in the drug release studies, showing lower melatonin release/skin permeation for the hydrogels prepared with the spray-dried nanocapsules as well as the synergistic effect of the strategies employed in this study (nanoencapsulation, spray-drying, and preparation of semisolid dosage forms). In addition, with a view to industrial production, spray-dried powders present the advantages of being less susceptible to microbiological contamination and more appropriate for storage and transport.

## Conclusions

Redispersible spray-dried melatonin-loaded nanocapsules were prepared using water-soluble excipients as drying adjuvants (lactose or maltodextrin). Different techniques showed the redispersion efficiency of spray-dried nanocapsules. Laser diffraction, photon correlation spectroscopy, and nanoparticle tracking analysis showed similar results. The hydrogels prepared with melatonin-loaded nanocapsules were able to control the release rate of melatonin as well as to delay its permeation across the pig skin. An additive effect of the nanoencapsulation, spray-drying, and incorporation in semisolid dosage forms explains the better control of the transdermal delivery of melatonin. This study represents a promising strategy for the use of spray-dried nanocapsules in the development of semisolid dosage forms for transdermal-controlled delivery nanomedicines.

## Abbreviations

D-NC-L: powders prepared with lactose; D-NC-M: powders prepared with maltodextrin; G-M: hydrogel prepared by adding directly the melatonin dispersed in water containing polysorbate 80; G-NC: hydrogel prepared by adding Carbopol 940® directly to the nanocapsule suspension; G-NC-L: hydrogel prepared by adding the spray-dried nanocapsules (prepared with lactose); G-NC-M: hydrogel prepared by adding the spray-dried nanocapsules (prepared with maltodextrin); MSC: model selection criterion; NC: melatonin-loaded polymeric nanocapsules; NTA: nanoparticle tracking analysis; PCS: photon correlation spectroscopy; RE: redispersion efficiency; SPAN: measure of the width of the distribution of particle size..

## Competing interests

The authors declare that they have no competing interests.

## Authors' contributions

CRDH carried out the preparation and characterization of nanoparticle formulations and *in vitro* permeation studies, and drafted the first version of the manuscript. TLD participated in the preparation and characterization of formulations. EAB preformed the nanoparticle tracking analysis and drafted the methodology. SRS design the powder redispersion methodology and helped in the preparation of the manuscript. RPR carried out the statistical analysis*/*mathematical modeling of data and helped in the interpretation and discussion of data. RCRB carried out the interpretation and discussion of results and wrote the final form of the manuscript. ARP participated in the design of experiments and interpretation of data. SSG conceived the study, participated in its design, coordination, result interpretations, and final approval of the version to be published. All authors read and approved the final manuscript.
